# Physical Diagnosis and Its Importance to the Dentist

**Published:** 1915-06

**Authors:** Walter C. Collins, John N. Gallagher


### PHYSICAL DIAGNOSIS AND ITS IMPORTANCE TO THE DENTIST.

READ BEFORE THE STUDENTS' DENTAL SOCIETY OF THE UNIVERSITY OF MICHIGAN, APRIL 5, 1915.

BY WALTER C. COLLINS AND JOHN N. GALLAGHER.

In considering such a topic we can not expect to cover the whole field, but we can discuss the means by which it is accomplished and its value to us as dentists. The word itself, means, the analysis of the case at hand, and the object of obtaining such information, is that we may proceed intelligently, either in a technical, or therapeutic manner. The detection of the phenomena of disease, is referred to as clinical diagnosis, and in this phase of diagnosis, much information may be obtained, although it may not be so definite as that obtained by more complete and detailed methods of diagnosis. By ordinary clinical means the disease may be located, and many times sufficient evidence can be obtained to justify accurate conclusions. In complete diagnosing the morbid conditions of the tissues should be observed, and the complications detected, and this introduces a new phase of diagnosis, namely, pathological diagnosis. Morbid conditions of tissues do not occur without a cause, and it should be our first aim to find and remove the cause, or at least aid nature in doing so, thus relieving the patient of the intruder, whatever it may be. So this necessitates the third phase of diagnosis, that of etiological diagnosis. Even this is too restricted a conception of diagnosis, for in some cases we must have conclusive evidence, hence we have the laboratory diagnosis. By this means definite knowledge may be obtained, and in many cases this is quite essential. For example, tumors may be impossible to properly diagnose as to their malignancy without the aid of the microscope and a blood test, especially if unusually abnormal. An examination of saliva may be very important, for instance, as when the carbohydrates are first started in digestion by the ptyalin in the saliva, and as ptyalin acts only in an alkaline media, the presence of excess acid in the saliva means impaired digestion of the carbohydrates, and this certainly should be corrected. And still further, the presence of a definite micro-organism is always indicative of a certain disease, consequently its presence conveys definite knowledge.

The methods of carrying on diagnosis may be divided into two subdivisions, namely, subjective and objective symptoms. The former are those which can be given by the patient, and are of great value. Examples of these symptoms are burning, a sense of fullness, itching, fatigue, etc. You can also ascertain as to whether the affliction is chronic or acute, and any of these, if understood in their nature and cause, certainly conveys useful information. On the other hand, there are those symptoms which may be observed by the operator, and if understood they are more definite in the information procured. The first sight of the patient, to the experienced practitioner, his general appearance, age, temperament, attitude, restlessness, size, muscular development, etc., all have a meaning which can be detected, but the most essential point we wish to bring out, is the importance of proper diagnosis to the dentist. We like to be spoken of, and rightly should be spoken of, as specialists in medicine, and if we are to maintain such a title, we must carry on more than a technical procedure. The rhinologist does not ask the general practitioner to instruct him about the nose and throat, neither does the eye specialist permit the general practitioner to dictate to him about the eye. If, then, we are to be professional men, and specialists in medicine, we should adopt methods that will give us definite knowledge of the sphere of our efforts. If we all, as professional men, work together, we can render the best services. If a patient comes to us who is suffering from a chronic alveolar abscess, or pyorrhea, and his systemic conditions are such that the tissues will not yield to the usual treatment, then it may be our duty to consult our medical confrere, or even to refer this patient to the medical man for a fuller diagnosis and help in our treatment. If the medical man finds systemic troubles, due to conditions in the mouth, it may be his duty to refer such patients to us for diagnosis, and in that way, we may render the best possible services to all our patients.

The importance of diagnostic ability is again brought out in connection with the treatment of children's teeth, It is a common thing for a child troubled with disease of the temporary teeth to be neglected by his parents. They do not consult the dentist about it as a rule until it is too late to remedy, and they then go to the dentist to have it extracted, in a large number of cases, and as a result, the little patient later suffers from malocclusion, or a defective breathing apparatus, defective speech, and even impaired health. If we are professional men we will not extract such a tooth, but we should carefully look into the nature of the disease and correct the condition as a conservative medical man would do, for the ultimate good of the patient. We are in duty bound to analyze the situation and do what is best for our patients, even though it means some inconvenience to ourselves and trouble to our patients.

Then is it always a question of how to perform an operation, or is it a question of what operation to perform? In answering this question let us consider what we are doing every day. We put in gold fillings from third molar to third molar, or we put in gold inlays which would be a credit to any practice, or we make artificial dentures that patients can wear, and we see abscesses yielding to our routine treatment. We find ourselves doing the technical routine with such uniformity in results that we too often forget to make careful examinations before starting an operation. We can't have a man of superior ability always at our side to diagnose our cases, and to tell us what to do when a troublesome case presents. We must learn not only how to make a complete diagnosis, but we should study every case carefully. We must keep our eyes open and know what to do and when. Then we shall know better than to extract temporary teeth prematurely, or do extensive operations on teeth with partially formed roots, or put crowns and bridges on diseased roots. We shall know what to do, and we shall put the right thing in the right place. Then our place as professional men will never be questioned, and technical work which would otherwise be good, will not result in disastrous failures.

